# Neuroprotective activity of novel phenanthrene derivative from *Grewia tiliaefolia* by in vitro and in silico studies

**DOI:** 10.1038/s41598-023-29446-7

**Published:** 2023-02-10

**Authors:** Ankita Rajput, Palvi Sharma, Nitish Kumar, Sarabjit Kaur, Saroj Arora

**Affiliations:** 1 Department of Botanical and Environmental Sciences, Guru Nanak Dev University, Amritsar, Punjab India; 2 Department of Pharmaceutical Sciences, Guru Nanak Dev University, Amritsar, Punjab India

**Keywords:** Computational biology and bioinformatics, Drug discovery

## Abstract

Medicinal plants possess range of phytochemicals accountable for their diverse biological activities. Presently, such compounds have been isolated from medicinal plants, characterized and evaluated for their pharmacological potential. In the present study, the efforts have been made to isolate the compound(s) from *Grewia tiliaefolia* Vahl., plant known for its ameliorative effect on brain related diseases such as anxiety, depression, cognitive disorders and Parkinson’s disease. Plant extract was subjected to isolation of compound(s) using column chromatography and isolated compound was characterized by NMR FTIR and LCMS. The isolated compound was novel with the IUPAC name of the compound is propyl 3-hydroxy-10,13-dimethyl-6,7,8,9,10,11,12,13,14,15,16,17-dodecahydro-3H-cyclopenta[a]phenanthrene-17-carboxylate, designated as A-1 and has not been reported before. A-1 was further evaluated for its antioxidant potential using in vitro antioxidant assays (2,2-diphenyl-1-picryl-hydrazyl-hydrate, DPPH assay and reducing power assay, RPA). Also, Acetylcholinesterase (AChE) inhibitory potential of A-1 and extract was analysed. Results showed that A-1 exhibited significantly higher antioxidant activity in both DPPH and RPA assay as compared to plant extract. In case of AChE inhibitory activity again, A-1 has shown significantly higher activity as compared to plant extract. In silico study was conducted to predict its action on proteins playing crucial role in neurological and neurodegenerative disorders such as gamma amino butyric acid (GABA) receptor and glutamate α amino-3-hydroxyl-5-methyl-4-isoxazolepropionic acid (Glu AMPA) receptor in epilepsy and AChE enzyme in Alzheimer’s diseases. The compound has shown interaction in following order: AChE > GABA receptor > Glu AMPA receptor. Further, molecular dynamic simulations and ADME studies of A-1 and AChE enzyme revealed that A-1 yielded good results in all parameters and hence can relieve Alzheimer’s like symptoms.

## Introduction

Humans have used plant resources for millennia, not only for food and shelter but also for health and well-being. Between 75 and 90 percent of the world's rural population still relies heavily on herbal extracts and preparations for the majority of their primary healthcare^1^. Medicinal plants contain a range of simple to complex secondary metabolites that may have therapeutic applications^2^. These metabolites are known to successfully prevent a number of chronic diseases such as cancer, alzheimer’s disease, epilepsy, diabetes and many more through a variety of mechanisms, including the reduction of oxidative stress, inhibition or modification of enzymes and receptors, interference with cellular signalling, and others^3–5^.

*Grewia* genus, consists of 150 species, a member of family Tiliaceae and is found in tropical and sub-tropical areas^6^. There are about 40 species of this genus in India, many of which have medicinal uses. *Grewia tiliaefolia* is one of these species^7^. In India, tribal people and traditional healers have utilised this plant as an aphrodisiac, expectorant, antipruritic, and astringent^8^. In vitro and in vivo tests conducted on this plant have revealed its anti-inflammatory, anti-cancer, and hepatoprotective properties. *G. tiliaefolia* exhibited anti-amyloidogenic and neuroprotective effect and also regulated glutamate transporters for mitigating the toxicity exerted by glutamate in Neuro-2a cells^9,10^. Given that *G. tiliaefolia* has neuroprotective potential, the objective of the present study was to isolate compound from the plant and evaluate it for antioxidant and AChE inhibitory activity. Further, its role was predicted using the suitable target proteins playing crucial role in the pathogenesis of neurological diseases. Disbalance of GABA receptors and Glu AMPA receptors play crucial role in the pathogenesis of epilepsy. Decreased GABA and increased Glu AMPA expression leads to GABA/Glutamate disbalance, causing seizures^11^. AChE can hydrolyse acetylcholine (ACh) and diminish the synaptic concentration more than expected. The level of AChE in the hippocampus and cortex of Alzheimer’s patients is higher than that of healthy individuals. Therefore, increasing ACh levels by blocking AChE is a key strategy for treating Alzheimer patients' cognitive impairment^12–14^. AChE inhibitors can attenuate neuronal damage and death from cytotoxic insults and therefore might affect Alzheimer Disease pathogenesis^15^. Hence, GABA receptor, Glu AMPA receptors and AChE were selected for in silico study.

## Material and methods

All methods were performed in accordance with the relevant guidelines/regulations/legislations required to conduct the study.

### Chemicals and reagents

Methanol, ammonium molybdate, and trichloroacetic acid were purchased from Sigma Aldrich (St. Louis, MO, USA). 2,2-diphenyl-1-picrylhydrazyl, ferric chloride, potassium ferricyanide, hydrogen peroxide, sodium monophosphate was procured from Himedia Laboratories Pvt. Ltd. (Mumbai, India). The rest of the reagents and chemicals used in the study were of analytical grade.

### Plant material and extraction

The dried aerial parts of *Grewia tiliaefolia* were procured from Sri Venkateshwara University, Tirupati, Andhra Pradesh. Dr. K. Madhava Chetty, Assistant Professor, Department of Botany, Sri Venkateshwara University, Tirupati, Andhra Pradesh, has done the authentication of plant material with Voucher no. 0420. The plant material was coarsely powdered and extracted successively using Soxhlet apparatus with hexane, chloroform, and methanol each for 72 h to separate fatty compounds. Each extract was filtered using Whatman filter paper, concentrated using a rotary evaporator (Bucchi Multivapor P-6, India), completely dried and stored at 4 °C for further use^16^. Among all extracts, *Grewia tiliaefolia* methanol extract has shown higher extractive yield (Supplementary file, S1).

### Isolation and characterization of compound

The *G. tiliaefolia* methanol extract was subjected to isolation using silica column. The column was then run with hexane (mobile phase) and fractions were collected. Compound was isolated from collected fractions and subjected to characterization using different techniques such as NMR (H^1^, C^13^, COSY, HMBC and HSQC), LCMS and FTIR for structure elucidation^17^.

### Antioxidant assays

#### DPPH assay

The free radical scavenging potential was analysed using a DPPH assay^18^. The different concentrations (25–400 µg/ml) of the samples were mixed with 0.1 mM DPPH solution and incubated at room temperature for 30 min in the dark room. Gallic acid was used as a reference compound. The absorbance was noted at 517 nm using a Biotek microplate reader (Agilent, Santa Clara, California). Inhibition potential was calculated using the following formula:

$${\text{Inhibition}}\,{\text{potential}} = \frac{{{\text{Ac}} - {\text{As}}}}{{{\text{Ac}}}} \times 100$$

Ac is the absorbance of the control, As is the absorbance of the sample.

#### RPA

The reducing power assay was conducted according to Benzie and Strain's technique (1996)^19^. Increasing concentrations (25–400 µg/ml) of samples (2 ml), phosphate buffer (2 ml, 0.2 M, pH 6.6), and potassium ferricyanide (2 ml, 1%) were mixed and then incubated for 20 min at 50 °C. Trichloroacetic acid (2 ml, 10%) was added to the reaction mixture. A volume of 2 ml from each of the reaction mixture was mixed with 2 ml of distilled water and 0.4 ml of 0.1% (w/v) ferric chloride in a test tube. After 10 min incubation, the absorbance was observed at 765 nm using a Biotek microplate reader (Agilent, Santa Clara, California). Ascorbic acid was used as standard. Inhibition potential was calculated using the following formula:

$${\text{Ferricyanide}}\,{\text{Reduction}}\,{\text{Potential}} = 1 - \left( {\frac{{{\text{Absorbance }}\,{\text{of}}\,{\text{ascorbic}}\,{\text{acid}} - {\text{Absorbance}}\,{\text{of}}\,{\text{sample}}}}{{{\text{Absorbance}}\,{\text{of}}\,{\text{ascorbic}}\,{\text{acid}}}}} \right) \times 100$$

### AChE inhibitory activity

AChE inhibitory activity of the extract and compound A-1 was investigated using Falé method. 7.5 μL of AChE solution containing 0.26 U/mL and 90 μL of 50 mmol/L Tris–HCl buffer, pH = 8.30 were mixed in a microwell plate and were incubated for 15 min. After that 142 μL of 3 mmol/L 5,5′-dithiobis(2-nitrobenzoic acid) and 22.5 μL of a solution of AChI (0.023 mg/mL) were added. The absorbance was taken at 405 nm when the reaction reached equilibrium using Biotek microplate reader (Agilent, Santa Clara, California). A control reaction was carried out using water in place of extract or compound A-1 and it was considered 100% activity^20^.

$${\text{Formula}}\,{\text{of}}\,{\text{Inhibition}}\,{\text{potential}},\left( {\text{\% }} \right) = 100 - \left( {\frac{A1}{{A0}}} \right) \times 100$$

A1: absorbance of the sample; A0: absorbance of the control reaction.

### In silico study

The isolated compound was docked with GABA receptor, AMPA receptor, and AChE which are involved in the pathogenesis of neurological disorders. Research Collaboratory for Structural Bioinformatics Protein Data Bank (RCSB PDB) website was used to download the PDB files of proteins and were loaded into the “*Prepare Receptor”* module of *Biosolveit Lead it* software version 2.3.2 (25.08.17). A co-crystallized ligand in the protein was used to provide the coordinates of the active binding site to the software, to generate a grid for docking the compound. The ligand was loaded into “*Define FlexX docking*” module of *Biosolveit Lead it* and was docked on respective proteins simultaneously. The binding energy (kcal/mol) and docking score of the protein–ligand complex was calculated from each pose generated using “*score a ligand with Hyde*” of *Biosolveit Lead it.* Biovia discovery studio visualizer 2020 was used to export and render the binding poses^21,22^.

### Molecular dynamic simulations

For generation of system of protein (PDB code: 1EVE) and ligand complex, a SPC model of solvent was selected with orthorhombic box shape with 5 × 5 × 5 dimensions by using “system builder” module of DE Shaw Desmond software. Later, Counter ions (Na + or Cl −) for neutralization were added, system’s concentration was set to 0.15 M and OPLS3e force field was applied to the system. This generated system was simulated for 50 ns by using “Molecular Dynamics” module of DE Shaw Desmond. A 5-stage minimization of model system was performed prior to final MD simulation. These stages are: (a) stage 1—simulates Brownian Dynamics NVT, T = 10 K, small time steps, and restraints on solute heavy atoms, 100 ps; (b) stage 2—simulates NVT, T = 10 K, small time steps, and restraints on solute heavy atoms, 12 ps; (c) stage 3—simulate NPT, T = 10 K, and restraints on solute heavy atoms, 12 ps; (d) stage 4—simulate, NPT and restraints on solute heavy atoms, 12 ps; (d) stage 5—simulate, NPT and no restraints, 24 ps; (e) stage 6—final simulation for 50 ns that covers about 200 K steps^22,23^.

### Lipinski rule of five and ADME studies

For calculation of Lipinski rule of five and ADME descriptors, molecular structure data was uploaded to two online data-servers. For Lipinski parameters, TargetNet website (http://targetnet.scbdd.com/home/index/) was used and for ADME studies, PreADME website (https://preadmet.webservice.bmdrc.org/) used^24,25^.

### Statistical analysis

The data was presented as mean ± SEM. Statistical data analysis was done by using one-way ANOVA followed by Tukey’s post hoc test by Graph Pad Prism version 5.0 and the significance level was *p < 0.05. Correlation coefficient was analysed using the Pearson’s coefficient of correlation between the AChE inhibitory activity and antioxidant activities (DPPH and RPA) of A-1 and r^2^ values were compared.

## Results

### Characterization of compound

**Figure 1:**
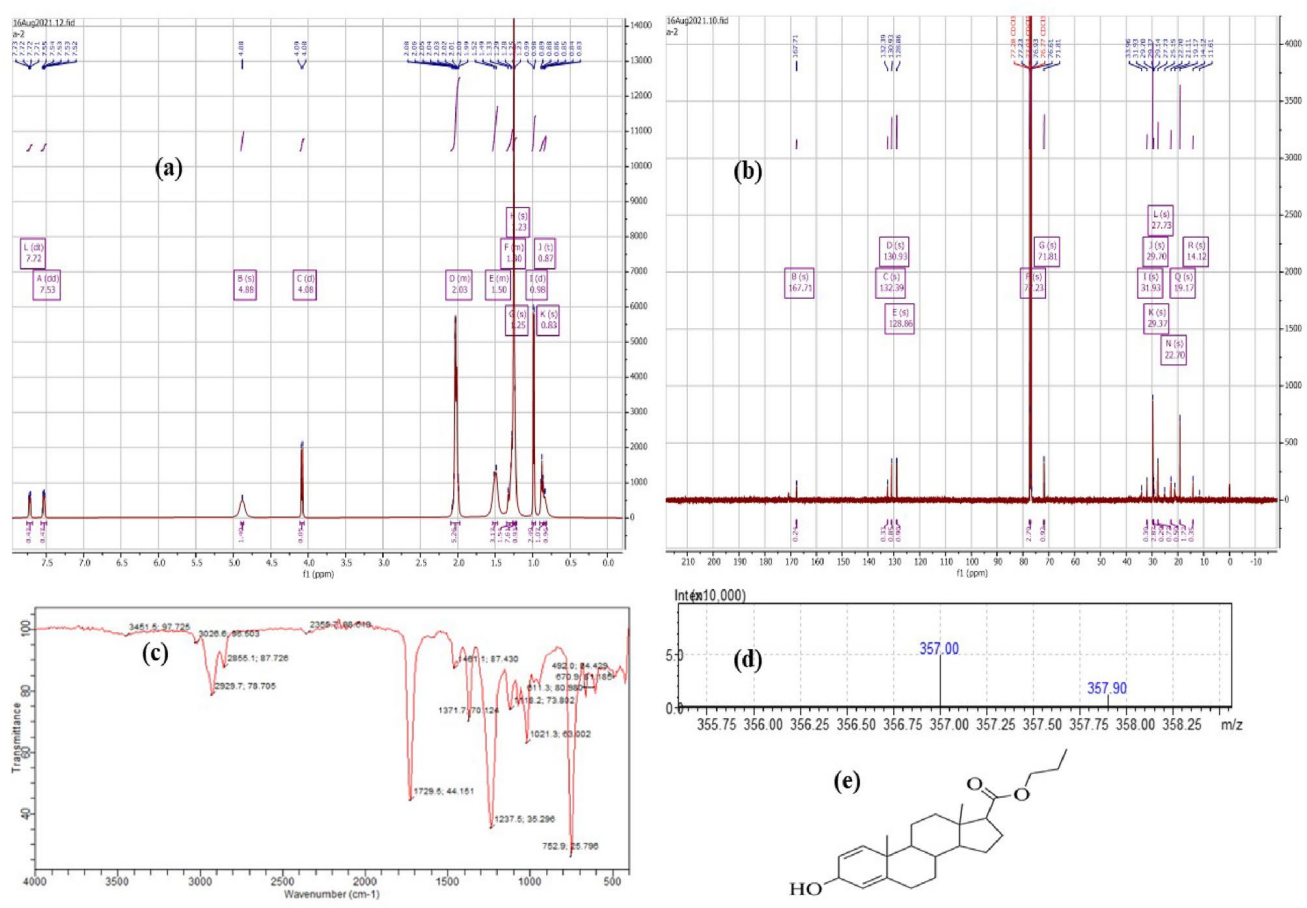
(**a**) ^1^H NMR spectra; (**b**) ^13^C NMR spectra; (**c**) FTIR spectra; (**d**) LCMS data; (**e**) Structure of A-1.

Compound was obtained as light green colored, solid substance about 37 mg approximately. ^1^H NMR (500 MHz, CDCl_3_) δ 7.72 (dt, *J* = 7.7, 3.8 Hz, 1H), 7.53 (dd, *J* = 5.8, 3.3 Hz, 1H), 4.88 (s, 1H), 4.08 (d, *J* = 6.7 Hz, 1H), 2.10–1.97 (m, 6H), 1.53–1.47 (m, 4H), 1.35–1.26 (m, 2H), 1.25 (s, 10H), 1.23 (s, 1H), 0.98 (d, *J* = 6.7 Hz, 4H), 0.87 (t, *J* = 6.7 Hz, 2H), 0.83 (s, 1H); ^13^C NMR (126 MHz, CDCl_3_) δ 167.71, 132.39, 130.93, 128.86, 77.28, 77.23, 77.03, 76.93, 76.77, 76.61, 71.81, 33.96, 31.93, 29.70, 29.37, 29.14, 27.73, 25.15, 22.70, 21.11, 19.17, 14.12, 11.61. Other spectra such as COSY, HMBC and HSQC are shown in supplementary file S2, S3 and S4. FTIR analysis: 3026.2:C-H (Aromatic), 2922.2 C-H (Alkane), 2855.1: C-H (Aromatic); 1729.5: C = O (Saturated); 670: C-H (Aromatic); 1729: C = O; 1237: C-O (Alkyl ester); 1461: C = C (Aromatic); 1371: O–H (Aromatic, phenol). LCMS (ESI +): m/z detected for C_23_H_34_O_3_ was 357.0 g/mol. IUPAC name of the compound: propyl 3-hydroxy-10,13-dimethyl-6,7,8,9,10,11,12,13,14,15,16,17-dodecahydro-3H-cyclopenta[a]phenanthrene-17-carboxylate. Structure of isolated compound (A-1) has been shown in Fig. 1. This is novel compound and has not been reported before.

### Antioxidant potential

#### DPPH assay

**Figure 2:**
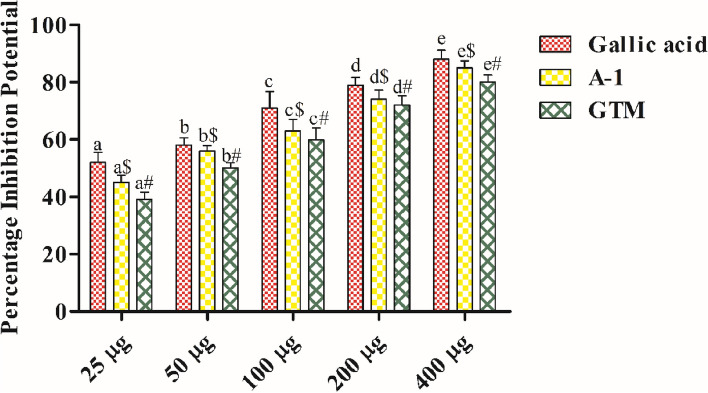
Percentage inhibition potential of Gallic acid, isolated compound and GTM. Data is presented as mean ± SEM. Abbreviations: *GTM G. tiliaefolia* methanol extract. Data sets with different alphabets differ significantly (p < 0.05) from each other.

DPPH is a free radical which is stable at room temperature. It is reduced in the presence of an antioxidant molecule and turns colorless from purple color. A-1 and *Grewia tiliaefolia* methanol extract had shown concentration dependant increase in inhibition potential. The inhibition potential was observed in the order *G. tiliaefolia* methanol extract < A-1 < Gallic acid. The IC_50_ values of Gallic acid, A-1 and *G. tiliaefolia* methanol extract were found to be 22.31 µg/ml, 36.08 µg/ml and 50.8 µg/ml respectively (Fig. 2).

#### RPA

**Figure 3:**
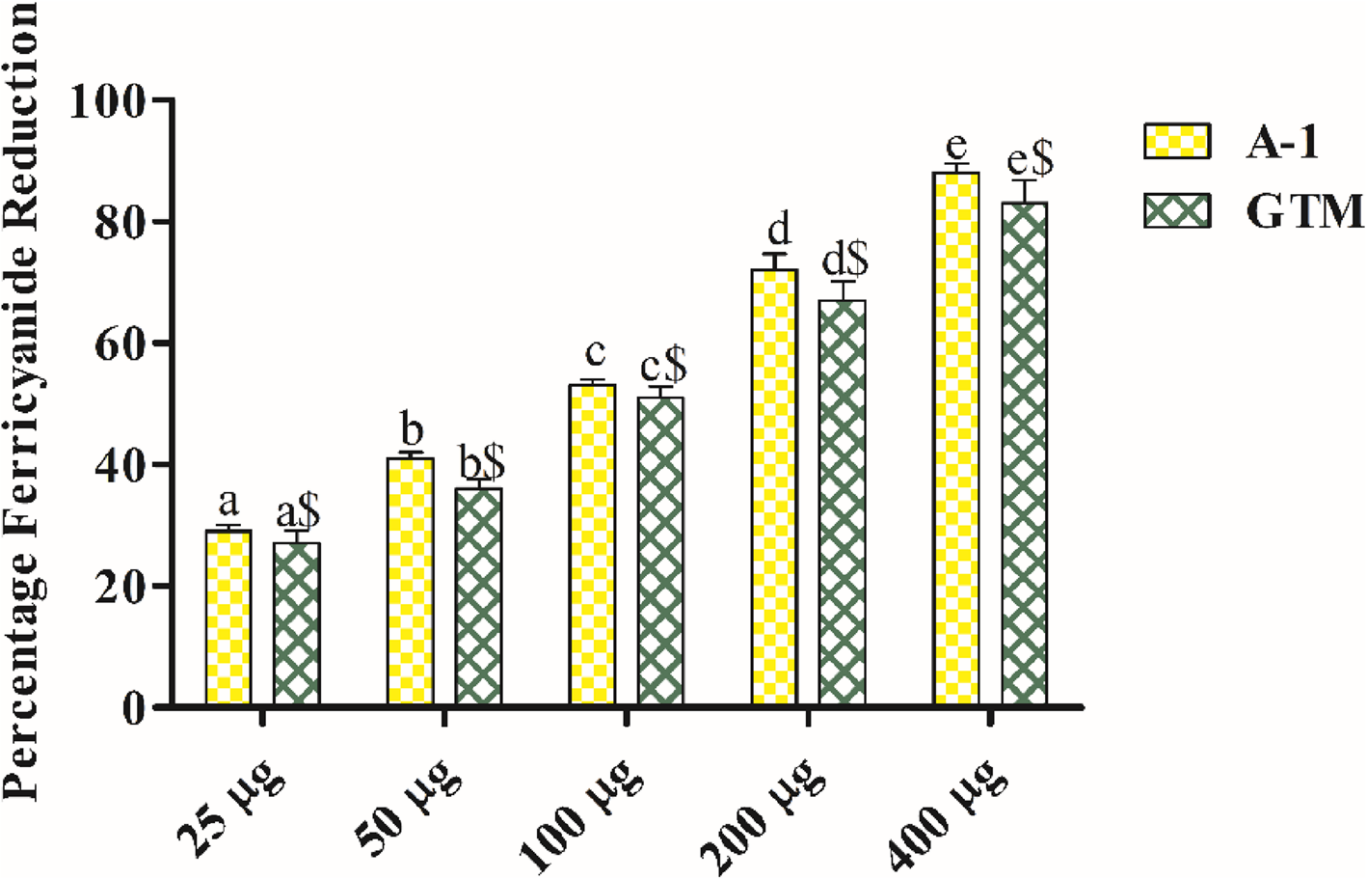
Reducing power assay of isolated compound and GTM. Data is presented as mean ± SEM. *GTM G. tiliaefolia* methanol extract. Data sets with different alphabets differ significantly (p < 0.05) from each other.

In this assay, the ferricyanide reduction potential was observed in the order: *G. tiliaefolia* methanol extract < A-1. The IC_50_ values of *G. tiliaefolia* methanol extract and A-1 were 149.82 µg/ml and 87.29 µg/ml respectively (Fig. 3).

### AChE inhibitory activity

**Figure 4:**
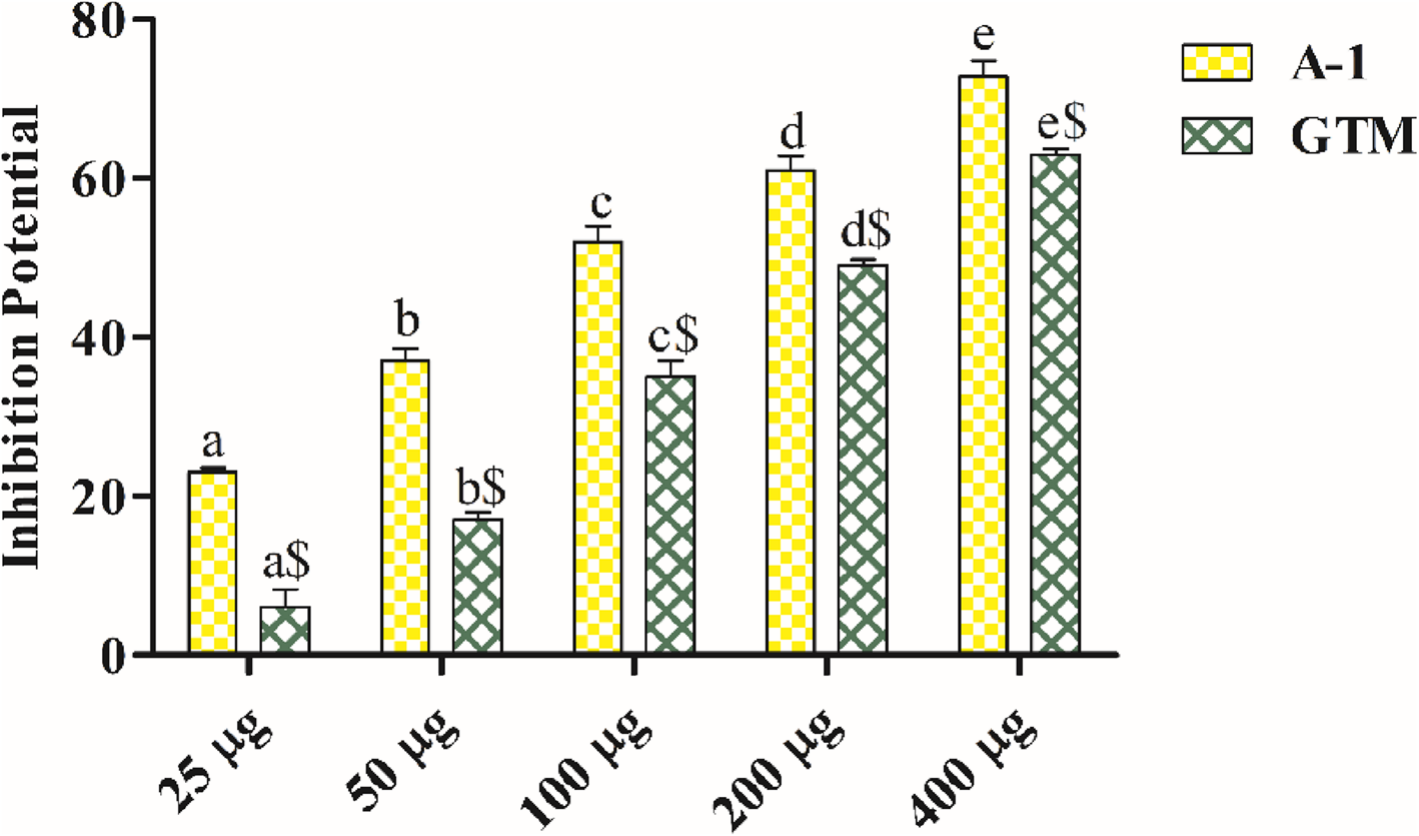
AChE activity of isolated compound and GTM. Data is presented as mean ± SEM. *GTM G. tiliaefolia* methanol extract. Data sets with different alphabets differ significantly (p < 0.05) from each other.

The AChE inhibitory activity of extract as well as the compound was evaluated. It was observed that both compound and extract have shown dose dependent increase in activity (Fig. 4). The compound had shown significantly higher inhibitory potential as compared to whole plant extract. The IC_50_ values for AChE inhibitory activity for A-1 and *G. tiliaefolia* methanol extract was found to be 104.81 µg/ml and 185.38 µg/ml, respectively.

### In silico study of compound

**Figure 5:**
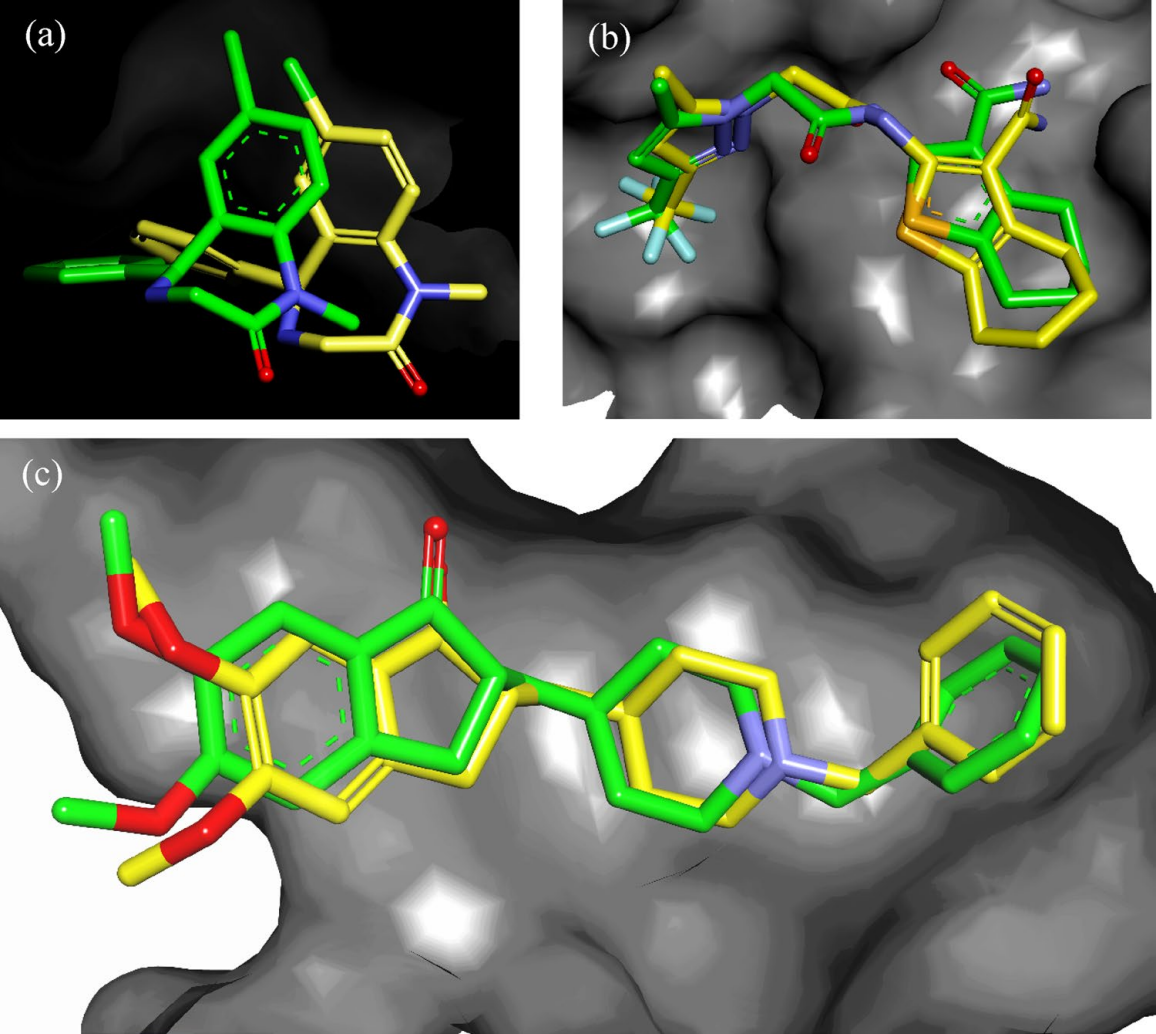
Overlapped poses of co-crystalized ligand (in green) and docked pose of same ligand (in yellow) in (**a**) GABA receptor (PDB code: 6X3X), (**b**) AMPA receptor (PDB code: 5YBF) and (**c**) AChE enzyme (PDB code: 1EVE) with their co-crystallized ligand i.e., Diazepam, HBT-1, Donepezil respectively.

Before docking the potent compound, we validated the docking protocol by re-docking the co-crystallized ligand of respective proteins and calculating their RMSD by using the crystallized pose as a reference. Upon comparison of the overlaid poses of the co-crystallized ligand and its docked pose, we observed no significant changes or deviation (depicted in Fig. 5) and the RMSD for all three proteins was within the statistical threshold of 3.0 Å, as reported in Table 1.

**Table 1:** Binding energies.

S. no	Interactions (protein X standard/compound)	PDB code	RMSD	Reference molecule	Binding energy (kcal/mol)	
					Reference molecule	A-1
1	GABA receptor	6X3X	2.7059	Diazepam	− 14	− 19
2	AMPA receptor	5YBF	1.2732	HBT-1	− 10	− 9
3	AChE	1EVE	0.8346	Donepezil	− 27	− 31

*GABA* Gamma-aminobutyric acid, *Glu AMPA* α-Amino-3-hydroxy-5-methyl-4-isoxazolepropionic acid, *AChE* Acetylcholinesterase, *kcal/mol* Kilocalories per mole.

Following validation, compound A-1 was docked onto the GABA receptor (PDB code: 6X3X), Glu AMPA receptor (PDB code: 5YBF), and AChE enzyme (PDB code: 1EVE), and their binding energies were compared to those of their co-crystallized ligands, Diazepam, HBT-1, and Donepezil, respectively. After docking the poses, we calculated their binding energies and evaluated their docked poses, as depicted in Fig. 5 and Table 1. The binding energies of the compounds were comparable to those of their reference molecules, and it was also noted that A-1 had significantly higher binding energies with the AChE enzyme as compared to other proteins (GABA and Glu AMPA receptors) as shown in Table 1. These high binding affinities towards AChE prompted us to investigate the time-dependent changes in the A-1-AChE complex using molecular dynamics simulations.

### Molecular dynamic simulations

**Figure 6:**
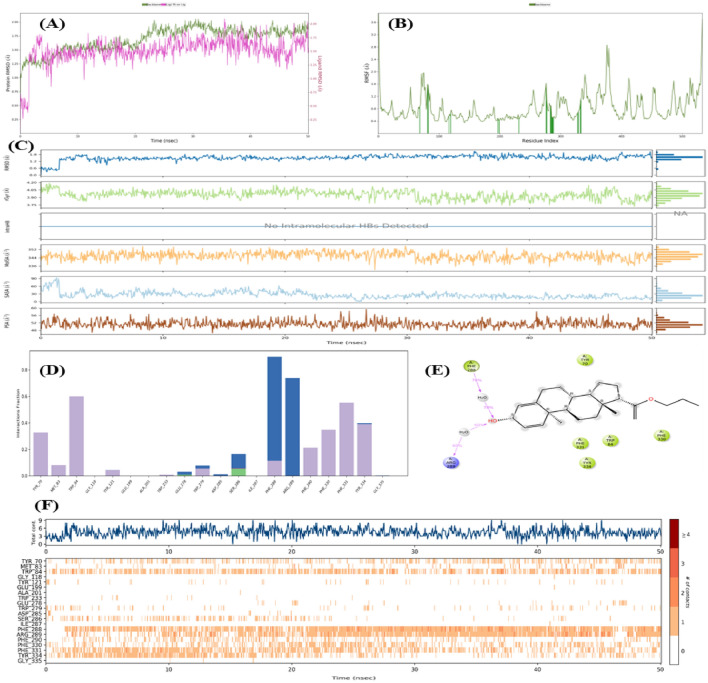
Molecular dynamic simulation of AChE enzyme (PDB code: 1EVE) and A-1 complex studied for 50 ns, where (**A**: RMSD protein ligand complex; **B**: RMSF plot of amino acids of proteins; **C**: RMSD, rGyr, Intra HB, MoISA, SASA and PSA plot of A-1; **D**: Histogram showing the type of protein ligand interaction, **E**: 2D interaction diagram of A-1 with protein’s amino acid residues and, **F**: Count of protein ligand contact during 50 ns).

To tackle the limitations of molecular docking i.e., no flexibility of protein structure, the whole protein ligand complex was simulated for 50 ns and time dependent stability of complex was studied as shown in Fig. 6. Complex was loaded into DE Shaw Desmond 2022 software and converted into a system that mimics the biological environment. This system was then simulated for 50 ns and after successful completion of simulation, various analytical parameters such as RMSD, RMSF, interaction fraction etc. were studied. Results showed that the RMSD of the protein and ligands were stable for 50 ns and value of RMSD was lesser than statistical limit of 3 Å. Moreover, RMSF value was also less than the statistical limit and hence there occurred no major conformational changes in the amino acids that are interacting with the ligand (shown as green solid vertical lines). Later on, studying the interaction fractions, it was found that protein has maximum of 9 interactions at a time with ligand and there was also addition of 5 water bridges between ligand and Glu278, Trp279, Asp285, Ser286, Phe288 Arg289 and Tyr334 amino acids while other amino acids were interacting via hydrophobic, hydrogen bonding and ionic interactions. Among all the amino acids of active binding site, Phe288 and Arg289 interacted the most with ligand i.e., interacted more than 60% of the time. In conclusion of MD studies, the protein and ligand complex are stable for 50 ns and such a low value of RMSD and RMSF predicts that the complex will be stable for much longer time span.

### Lipinski parameters and ADME studies

Structure of isolated molecule was uploaded to PreADMET website (https://preadmet.webservice.bmdrc.org/) and all the ADME related parameters were calculated. Results were summarized in Table 2 and showed that the compound has higher affinities to cross blood brain barrier (BBB) with a value of 11.28 which is quite higher than the threshold value of 2.0. This BBB penetration power is beneficial for the compound to reach the AChE enzyme in the brain and inhibits its activity and bring relief to an Alzheimer patient. The compound is strongly bound to plasma protein with 100% affinity. Human intestinal absorption (HIA) of the compound is more than 90% indicating good oral absorption and the compound is not an inhibitor of CYP_2D6_ enzyme, therefore there is no drug-gene interaction. Moreover, on studying the Lipinski’s parameters as shown in Table 2, the compound possesses drug-like properties as it obeys Lipinski’s rule of five. All the five parameters that are hydrogen bond donor (HBD), Hydrogen bond Acceptor (HBA), Molecular refractivity (MR), Molecular weight (MW) and LogP values were within the limits i.e. < 5, < 10, 40–130, 180–480 and, − 0.4 to + 5.6 respectively.

**Table 2:** Summary of lipinski and ADME analysis.

**ADME evaluation**	
BBB	11.28
CYP_2D6_ inhibition	Non
HIA	97.81
Plasma protein binding	100
Aqueous solubility mg/l	0.81
**Lipinski’s parameters**	
HBD	1
HBA	7
LogP	5.6
MR	109.3
MW	356.5

*BBB* blood brain barrier, *CYP* cytochome P, *HIA* human intestinal absorption, *HBD* hydrogen bond donor, *HBA* hydrogen bond acceptor, *MR* molecular refractivity, *MW* molecular weight.

Lipinski and ADME studies indicated that the compound obeyed all the parameters and has drug like character. Hence this compound can be further evaluated via in vivo and in vitro studies.

### Correlation coefficient analysis

Pearson's coefficient of correlation was used to examine the correlation between AChE inhibitory activity and antioxidant activities (DPPH and RPA). These results have showed that the r^2^ value of the datasets was more than 0.90 as shown in Table 3. This indicates a significant correlation between them. Moreover, the datasets were following the same trend i.e., with an increase in the concentration of the compound there was an increase in the activity. From this it can be concluded that A-1 is a potent AChE inhibitor as well as an effective antioxidant also. Hence, this duple property of A-1 can be useful in drug development for neurological disorders.

**Table 3:** Correlation analysis using Pearson’s coefficient of correlation.

Activity	Pearson’s coefficient (r^2^)
AChE and DPPH	0.97
AChE and RPA	0.96

*AChE* acetylcholinesterase inhibitory, *DPPH* 2,2-diphenyl-1-picryl-hydrazyl-hydrate, *RPA* reducing power assay.

## Discussion

Propyl 3-hydroxy-10,13-dimethyl-6,7,8,9,10,11,12,13,14,15,16,17-dodecahydro-3H-yclopenta[a]phenanthrene-17-carboxylate, a cholesterol like moiety, designated as A-1 was isolated from *Grewia tiliaefolia.* A-1 has shown significantly higher antioxidant activity in DPPH and reducing power assay and higher AChE inhibitory activity as compared to plant extract. Also, the docking study have revealed that compound interacts with the proteins involved in the pathophysiology of epilepsy and other neurodegenerative disorders.

Isolation of compounds is of prime importance in the field of drug research and development^26^. For characterization and structure elucidation, different spectral techniques were used such as NMR, FTIR and LCMS. NMR (proton and Carbon) which indicates the number of carbon and hydrogen atoms present in the compound HSQC NMR spectra informs about the proton-carbon single bond correlations, where the protons lie along the observed F2 (X) axis and the carbons are along the F1 (Y) axis^27^. COSY NMR spectra appraises about the proton-proton bonds^28^. HMBC NMR spectra gives detail about 1H/13C multiple-bond connectivity^29^. FTIR analysis implicates the different functional groups present in the compound^30^. LCMS informs about the mass of compound^31^. Collectively, the whole information is gathered to elucidate the structure. *G. tiliaefolia* methanol extract was subjected to isolation using column chromatography. Compound was isolated and characterized using spectral analysis, such as NMR, FTIR and LCMS. Gathering all the information structure was elucidated. IUPAC name of the compound is Propyl 3-hydroxy-10,13-dimethyl-6,7,8,9,10,11,12,13,14,15,16,17-dodecahydro-3H-cyclopenta[a]phenanthrene-17-carboxylate.

Reactive oxygen species (ROS) that are produced as a result of oxidative stress in the biological system can cause damage to biological macromolecules, which can make the pathological condition at the cellular level more apparent^32^. Such a pathogenic condition which develops due to this imbalance between the antioxidant defence system and reactive oxygen species generation, is fundamental in the development of neurological illnesses as well as a number of other disorders^33^. Antioxidant analysis is a preliminary step in the pharmacological evaluation of drugs or extracts^34^. Antioxidant activity of extract and A-1 was analyzed by DPPH and RPA assay. In DPPH assay, A-1 exhibited higher activity as compared to extract. In RPA similar pattern was observed. A-1 was found to be stronger antioxidant as compared to plant extract.

AChE is responsible for the breakdown of ACh in the synaptic area which inhibits the nerve impulses^12^. ACh plays a significant part in brain processes which all gets affected after its level decline^35^. Low levels of ACh have been discovered to be associated with neurological disorders that eventually cause cognitive decline^35^. Natural cholinesterase inhibitors are commonly used for the treatment of neurological disorders so as to maintain the ACh level in brain^13,14^. Keeping this in mind AChE inhibitory activity of isolated compound was evaluated. It was observed that compound has shown significantly higher activity as compared to plant extract.

Pharmaceutical industry now has a great opportunity to uncover new prospective drug targets thanks to sophisticated in silico methods, which has a direct impact on the efficiency and duration of clinical trials for finding novel drug targets^36,37^. In silico study was conducted to envisage the interaction of isolated compound with selected target proteins which play important role in the pathogenesis of the neurological disorders such as epilepsy and Alzheimer’s disease. The results have shown that compound was found to interact with all the target proteins but has shown highest binding energy with AChE. The order of interaction of compound with target proteins is AChE > GABA receptor > Glu AMPA receptor.

Although docking results revealed that compound showed maximum binding energy with AChE, but alone docking study is not sufficient to predict the success of molecule as a drug. Hence molecular dynamic simulation and Lipinski and ADME studies were performed to further evaluate its dynamics within the body. Results from MD simulations studies indicated that the protein and ligand complex are stable for 50 ns and low values of RMSD and RMSF implicates that the complex will be stable for much longer time span.

The research in the field of phytochemicals has focused on investigation of natural compounds responsible for antioxidative and neuroprotective properties that can also be useful for neurodegenerative disorders such as Alzheimer’s diseases^38^. It is important to stimulate the cholinergic receptors in the CNS or enhance the prolonged production of ACh in the synaptic cleft with the help of such active constituents that could retard the activities of AChE enzyme in the neuronal system. Compound can be considered as strong inhibitor, if inhibition of enzyme by that compound is 60% or more^39^. In our study, it was observed that A-1 has shown significantly higher AChE inhibitory activity and also molecular dynamic simulation studies and Lipinski/ADME studies further revealed that molecule showed good results in all parameters and can serve as Anti-Alzheimer drug, although appropriate in vitro and in vivo model are required to confirm its success at preclinical level.

## Conclusion

The A-1 was novel compound. A-1 had shown significantly higher antioxidant activity as well as AChE inhibitory activity and stronger interaction with proteins involved in epilepsy and other neurodegenerative disorders, especially AChE enzyme. Further, this compound formed stable complex with AChE and remained stable for longer period, capable of crossing the BBB and obeys all drug like characteristics. This study provides the scientific basis for its possible pharmacological role as neuroprotective agent. Further studies are required to confirm the efficacy of A-1 against the epilepsy and Alzheimer’s disease at the preclinical and clinical level.

## Supplementary Information

Supplementary Information.

## Data Availability

The datasets used and/or analyzed during the current study will be available from the corresponding author on reasonable request.

## Abbreviations

GABA: Gamma amino butyric acid
Glu AMPA: Glutamate α amino-3-hydroxyl-5-methyl-4-isoxazolepropionic acid
AChE: Acetylcholinesterase
DPPH: 2,2-Diphenyl-1-picrylhydrazyl
RPA: Reducing power assay
RCSB PDB: Research Collaboratory for Structural Bioinformatics Protein Data Bank
SEM: Standard error of mean
GTM: *Grewia tiliaefolia* Methanol extract
NMR: Nuclear magnetic resonance
HMBC: Heteronuclear multiple-bond coherence
HSQC: Heteronuclear single-quantum coherence
COSY: ^1^H–^1^H correlation spectroscopy
FTIR: Fourier transform infrared
LCMS: Liquid chromatography mass spectrometry
NVT: Constant number (N), volume and temperature
NPT: Constant number (N), pressure and temperature
BBB: Blood brain barrier
CYP: Cytochome P
HIA: Human intestinal absorption
HBD: Hydrogen bond donor
HBA: Hydrogen bond acceptor
MR: Molecular refractivity
MW: Molecular weight
RMSD: Root mean square deviation
RMSF: Root mean square fluctuation
MD: Molecular dynamics
