# Supplementary material for: Neuroprotective activity of novel phenanthrene derivative from Grewia tiliaefolia by in vitro and in silico studies
Source: Sci Rep. 2023 Feb 10;13:2444. doi: 10.1038/s41598-023-29446-7 (PMC9918530; doi:10.1038/s41598-023-29446-7)
Supplement: Supplementary file 1 — Supplementary Information. [file 41598_2023_29446_MOESM1_ESM.docx]

**S1. Extractive yield of *G. tiliaefolia* extracts**

| **S. No.** | **Extracts** | **Extractive yield** |
| --- | --- | --- |
| **1** | *G. tiliaefolia* hexane extract | 0.26% |
| **2** | *G. tiliaefolia* chloroform extract | 1.62% |
| **3** | *G. tiliaefolia* methanol extract | 13% |

**S2. COSY:** ^1^H-^1^H Correlation Spectroscopy

**S3. HSQC:** Heteronuclear Single-Quantum Coherence

**S4. HMBC**: Heteronuclear Multiple-Bond Coherence
